# Gynecologic tumor board: a radiologist’s guide to vulvar and vaginal malignancies

**DOI:** 10.1007/s00261-021-03209-2

**Published:** 2021-08-25

**Authors:** Lucy Chow, Brian Q. Tsui, Simin Bahrami, Rinat Masamed, Sanaz Memarzadeh, Steven S. Raman, Maitraya K. Patel

**Affiliations:** 1 Department of Radiological Sciences, David Geffen School of Medicine, University of California, Los Angeles, 757 Westwood Plaza, Suite 1638, Los Angeles, CA 90095 USA; 2 Department of Obstetrics and Gynecology, David Geffen School of Medicine, University of California, Los Angeles, Los Angeles, CA 90095 USA; 3 UCLA Eli and Edythe Broad Center of Regenerative Medicine and Stem Cell Research, University of California Los Angeles, Los Angeles, CA 90095 USA; 4 Molecular Biology Institute, University of California Los Angeles, Los Angeles, CA 90095 USA; 5 The VA Greater Los Angeles Healthcare System, Los Angeles, CA 90073 USA; 6 UCLA Jonsson Comprehensive Cancer Center, University of California Los Angeles, Los Angeles, CA 90095 USA

**Keywords:** Vulvar cancer, Vaginal cancer, FIGO staging system, Pelvic imaging

## Abstract

Primary vulvar and vaginal cancers are rare female genital tract malignancies which are staged using the 2009 International Federation of Gynecology and Obstetrics (FIGO) staging. These cancers account for approximately 2,700 deaths annually in the USA. The most common histologic subtype of both vulvar and vaginal cancers is squamous cell carcinoma, with an increasing role of the human papillomavirus (HPV) in a significant number of these tumors. Lymph node involvement is the hallmark of FIGO stage 3 vulvar cancer while pelvic sidewall involvement is the hallmark of FIGO stage 3 vaginal cancer. Imaging techniques include computed tomography (CT), positron emission tomography (PET)-CT, magnetic resonance imaging (MRI), and PET-MRI. MRI is the imaging modality of choice for preoperative clinical staging of nodal and metastatic involvement while PET-CT is helpful with assessing response to neoadjuvant treatment and for guiding patient management. Determining the pretreatment extent of disease has become more important due to modern tailored operative approaches and use of neoadjuvant chemoradiation therapy to reduce surgical morbidity. Moreover, imaging is used to determine the full extent of disease for radiation planning and for evaluating treatment response. Understanding the relevant anatomy of the vulva and vaginal regions and the associated lymphatic pathways is helpful to recognize the potential routes of spread and to correctly identify the appropriate FIGO stage. The purpose of this article is to review the clinical features, pathology, and current treatment strategies for vulvar and vaginal malignancies and to identify multimodality diagnostic imaging features of these gynecologic cancers, in conjunction with its respective 2009 FIGO staging system guidelines.

## Introduction

Primary vulvar and vaginal cancers are rare, composing approximately 5-6% and 2%, respectively, of all female genital tract malignancies and account for approximately 2,700 number of deaths annually in the USA [1–3]. Although the vulva and vagina are located anatomically adjacent to each other and cancers in these locations have overlapping clinical populations, they are distinct tumors with different staging systems and treatments. In addition, women with vulvar or vaginal cancer may be asymptomatic and the disease diagnosed incidentally.

Primary vulvar cancers are staged surgically and clinically whereas primary vaginal cancers are staged clinically, both using the 2009 International Federation of Gynecology and Obstetrics (FIGO) staging system (Tables 1 and 2) [4]. An exemption is melanoma, which is staged clinically. The FIGO system and the American Joint Committee on Cancer (AJCC) TNM staging system are essentially the same, taking into account the size and extent of the tumor, lymph node involvement, and metastasis to distant sites. While physical examination may detect these malignancies and can evaluate the visible local disease extent, it cannot detect lymphadenopathy and metastatic disease reliably. Although the data on the role of imaging in diagnosis and staging of these gynecologic malignancies are limited, there is an increasing need to determine the pretreatment extent of disease given the move toward more tailored surgical approaches and use of neoadjuvant chemoradiation therapy to reduce morbidity. The FIGO staging system allows the use of imaging modalities such as CT, MR, and PET-CT to guide therapy which can help determine the full extent of disease for radiation planning and also help evaluate the response to treatment.

**Table 1:** FIGO and TNM Staging for Vulvar Cancer

FIGO stage	TNM stage	Tumor size	Lymph node involvement	Organ involvement	Distant metastases
1A	T1a N0 M0	≤2 cm and ≤ 1 mm stromal invasion	None	Vulva and/or perineum	None
1B	T1b N0 M0	>2 cm and > 1 mm stromal invasion	None	Vulva and/or perineum	None
2	T2 N0 M0	Any size	None	Anus, lower vagina, or lower urethra	None
3A	T1 or T2 N1 M0	Any size	1 inguinofemoral node > 5 mm or 1–2 nodes < 5 mm	Anus, lower vagina, or lower urethra	None
3B	T1 or T2 N2a or N2b M0	Any size	>2 inguinofemoral node > 5 mm or >3 nodes < 5 mm	Anus, lower vagina, or lower urethra	None
3C	T1 or T2 N2c M0	Any size	Positive nodes with extracapsular spread	Anus, lower vagina, or lower urethra	None
4A	T1 or T2 N3 M0 OR T3 Any N M0	Any size	Fixed/ulcerated inguinofemoral nodes Any N	Anus, lower vagina, or lower urethra Rectum, upper vagina, upper urethra, bladder, or pelvic bone	None
4B	Any T Any N M1	Any size	Any N	Any T	Yes Most commonly pelvic nodes or lung

**Table 2:** FIGO and TNM staging for vaginal cancer with MRI characteristics for each stage

FIGO stage	TNM staging	Tumor size	Lymph node involvement	Organ involvement	Distant metastases	MRI
1	T1a N0 M0	<2 cm	None	Vagina	None	Intact T2-hypointense vaginal wall
	T1b N0 M0	>2 cm	None	Vagina	None	
2	T2a N0 M0	<2 cm	None	Invades paravaginal tissue but not to pelvic sidewall	None	Disrupted T2-hypointense vaginal wall
	T2b N0 M0	>2 cm	None	Invades paravaginal tissue but not to pelvic sidewall	None	
3	T1, T2, or T3 N1 M0 OR T3 N0 M0	Any size Any size	Inguinal or pelvic nodes None	Invades pelvic sidewall, and/or lower vagina, and/or causing hydronephrosis Invades pelvic sidewall, and/or lower vagina, and/or causing hydronephrosis	None None	Abnormal T2-hyperintense pelvic musculature
4A	T4 Any N M0	Any size	Any N	Bladder, rectum, or extend beyond pelvis	None	Disrupted T2-hypointense bladder or rectal wall; abnormal enhancement of adjacent organs
4B	Any T Any N M1	Any size	Any N	Any T	Yes Lung, liver, or bones	Distant organ metastases (lungs and liver are most common)

The objective of this article is to discuss the background, clinical presentation, and risk factors of these two malignancies. Also, the FIGO staging guidelines for vulvar and vaginal cancers will be reviewed along with relevant imaging characteristics across multiple modalities. Finally, we will examine the imaging findings that guide treatment, assess treatment response, and delineate potential complications.

## Vulvar cancer

Vulvar cancer is a rare malignancy with approximately 6,200 new cases in 2018 [5]. The disease primarily affects post-menopausal women, with the median age at diagnosis of 68 years old [6]. In women older than 60 years, the disease is associated with chronic inflammation and lichen sclerosus [7]. In younger women, the disease is strongly associated with human papillomavirus (HPV) 16 or 18 and vulvar intraepithelial neoplasia (VIN). Although incidence rates have increased in women under 60 years old, high HPV vaccination coverage is expected to reverse the effect on the number of vulvar cancer cases in the vaccinated cohorts [8]. Other risk factors include smoking, HIV, and other genital cancers such as cervical cancer and melanoma.

Women clinically present with symptoms such as pruritus, pain, palpable mass, or bloody discharge. Patients may also be asymptomatic but on exam are noted to have a lesion in the vulva. The lesion may appear as an area of skin thickening or discoloration, raised plaque, ulceration, or palpable lump. A majority of vulvar cancers are diagnosed in the early stages, with 59% of vulvar cancer with localized disease. Approximately 30% of vulvar cancers present with spread to regional lymph nodes and only 6% of vulvar cancers present with distant metastases [6].

### Pathology

Squamous cell carcinoma (SCC) is the most common histologic subtype of vulvar cancer and comprises 70% of cases, with a bimodal distribution affecting women younger than 50 years old and women over 70 years old [9]. SCC arises from epidermal squamous cells and often is associated with vulvar intraepithelial neoplasia (VIN). There are several subtypes of squamous cell carcinoma. The most common is the keratinizing subtype which develops in older women and not associated with HPV. Less common subtypes include basaloid and warty subtypes which are seen in younger women with HPV. Verrucous carcinoma is an uncommon subtype that is slow-growing and correlated with a good prognosis.

The remaining cell types include adenocarcinoma, melanoma, basal cell carcinoma, and sarcoma. Adenocarcinomas most commonly arise from the Bartholin glands at the vaginal introitus. Paget’s disease of the vulva is an adenocarcinoma which mainly affects post-menopausal Caucasian women and originates from vulvar apocrine gland-bearing skin cells. Basal cell carcinoma is generally a slow-growing lesion, primarily located on the labia majora.

Finally, vulvar sarcomas include subtypes such as leiomyosarcomas, epithelioid sarcomas, malignant rhabdoid tumor, and rhabdomyosarcomas. These can be aggressive, have high rates of metastasis, recurrences, and high mortality rate [10]. Unlike other cancers of the vulva, vulvar sarcomas can occur in women of any age, including in childhood.

### Anatomy

**Fig. 1:**
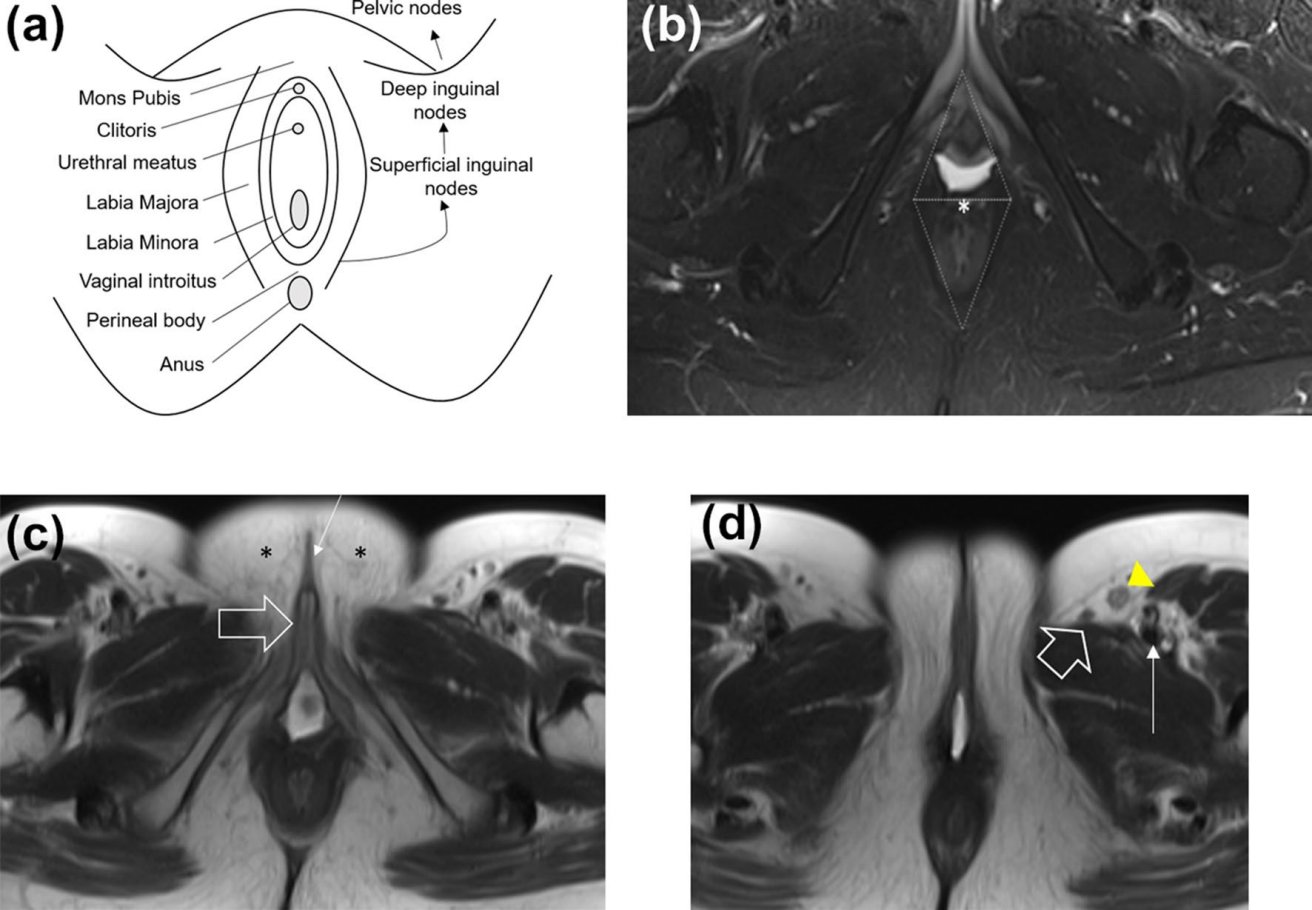
Relevant anatomy of the vulva. **a** Schematic of the vulva anatomy and lymphatic drainage pattern. The vulva is composed of the following components: mons pubis, clitoris, labia majora, labia minora, vaginal introitus, and the perineal body. The pattern of lymphatic drainage from the vulva occurs through a stepwise fashion from the superficial inguinal nodes, to the deep inguinal nodes, and then to the pelvic nodes. **b** Axial T2-weighted fat-saturated MR image shows the perineal body (asterisk), the urogenital triangle (anterior triangle), and anal triangle (posterior triangle). **c** Axial T2-weighted MR image shows the clitoris (large arrow), labia minora (small arrow), and labia majora (asterisks). **d** Axial T2-weighted image shows a superficial inguinal lymph node (yellow arrowhead) located anterolateral to the great saphenous vein (large arrow) and anterior to the femoral vessels (small arrow)

The vulva is a diamond-shaped soft tissue structure located external to the vagina. The vulva is composed of the following components: labia majora, labia minora, mons pubis, clitoris, vaginal introitus, and the perineal body (Fig. 1). Vulvar malignancies mainly affect the labia, with the labia majora composing about 50% of cases and the labia minora comprising about 20% of cases [11]. The vulva is located in close proximity to the distal vagina, distal urethra, and anal sphincter, which are potential common sites for local invasion.

### Patterns of spread

Vulvar cancer is primarily spread via the lymphatic system. The incidence of inguinofemoral lymph node metastasis is correlated with the depth of stromal invasion, with less than 1% lymph node involvement when the depth of invasion is less than 1 mm, and greater than 8% lymph node involvement when the depth of invasion is more than 1 mm, hence necessitating the need for lymph node dissection [12]. The incidence of deeper iliac chain lymph node metastases is 5%. Hematogenous spread to the lung and bones occur in approximately 4% of patients [13].

The pattern of lymphatic drainage from the vulva occurs through a stepwise fashion from the superficial inguinal nodes (most common), to the deep inguinal nodes, and then to the pelvic nodes. The superficial inguinal nodes are located anterior to the inguinal ligament, the superficial femoral vessels, and the saphenous veins. The deep inguinal nodes are located within the femoral sheath and medial to the common femoral vein. The deep inguinal nodes then drain to the iliac and paraaortic lymph nodes, which are both considered distant metastasis. Metastasis to the contralateral groin or pelvic nodes is rare in the absence of ipsilateral groin metastases. Also, metastasis to the deep inguinal nodes without the involvement of the superficial inguinal nodes has been reported, but this is also rare [14].

### Imaging

#### CT

CT is useful for detection of lymphadenopathy, determining bladder or rectal invasion and identifying distant metastases, including pulmonary and bony metastases. In addition, CT is used in planning radiation treatment. However, a limitation of CT is its inability to determine local tumor staging due to low soft tissue contrast resolution. Even with contrast-enhanced CT, the vulva may not be well delineated.

#### MRI

The benefits of MRI imaging include having superior contrast resolution and soft tissue delineation of the primary vulvar tumor as well as being the most sensitive modality for detecting lymph node involvement. MRI is the study of choice for local invasion and treatment response. Small field-of-view images of the pelvis allow for improved anatomy detail and tumor delineation, whereas large field-of-view images offer better detection of lymphadenopathy and bony metastasis. In a 2002 study, Hawnaur et al. determined that MRI had a sensitivity of 89% and specificity of 91% when determining the presence of metastatic lymphadenopathy [15]. In a 2010 retrospective study, Kataoka determined that contrast-enhanced MRI was useful in improving the accuracy of vulvar cancer staging in 85% of a cohort of 20 patients who underwent MRI before surgery [16].

#### PET-CT

PET-CT is useful with radiation therapy planning and as a supplementary imaging tool to lymphatic mapping and sentinel lymph node dissection [17]. In addition, PET-CT is helpful with assessing response to neoadjuvant treatment before surgery and is useful for determining prognosis and patient management. In a 2016 study, Robertson et al. determined that a clinician’s prognostic impression changed in half of the cases following information found on PET-CT. The imaging results helped determine management in 36% of cases, including opting for additional biopsy or treatment in lieu of watchful waiting [18]. On PET-CT, the primary vulvar lesion and the involved lymph nodes will demonstrate FDG avidity.

PET-CT may help detect involved regional hypermetabolic lymph nodes and distant metastases. However, PET-CT has limited value in detecting lymph node metastases less than 5 mm and necrotic lymph nodes. Furthermore, inflammatory lymph nodes can be false-positive on PET-CT [19].

#### PET-MRI

More recent technology of PET-MRI combines the diagnostic advantages of detailed high-resolution anatomic information from MR with the metabolic information from PET. In a 2018 study comprising a cohort of seventy-one women with gynecologic cancers, Sawicki et al. determined that PET-MRI correctly identified 100% of vulvar cancer recurrences and 84% vaginal cancer recurrences compared to MRI alone [20]. PET-MRI was able to recognize more pelvic recurrences and distant metastases compared to MRI alone.

A potential limitation of fused PET-MRI would be misregistration that can occur between the independently acquired PET and MRI studies. However, newer integrated PET-MRI systems allow for simultaneous acquisition of PET and MRI data, which decreases this.

### FIGO classifications and imaging features

#### FIGO stage 1

**Fig. 2:**
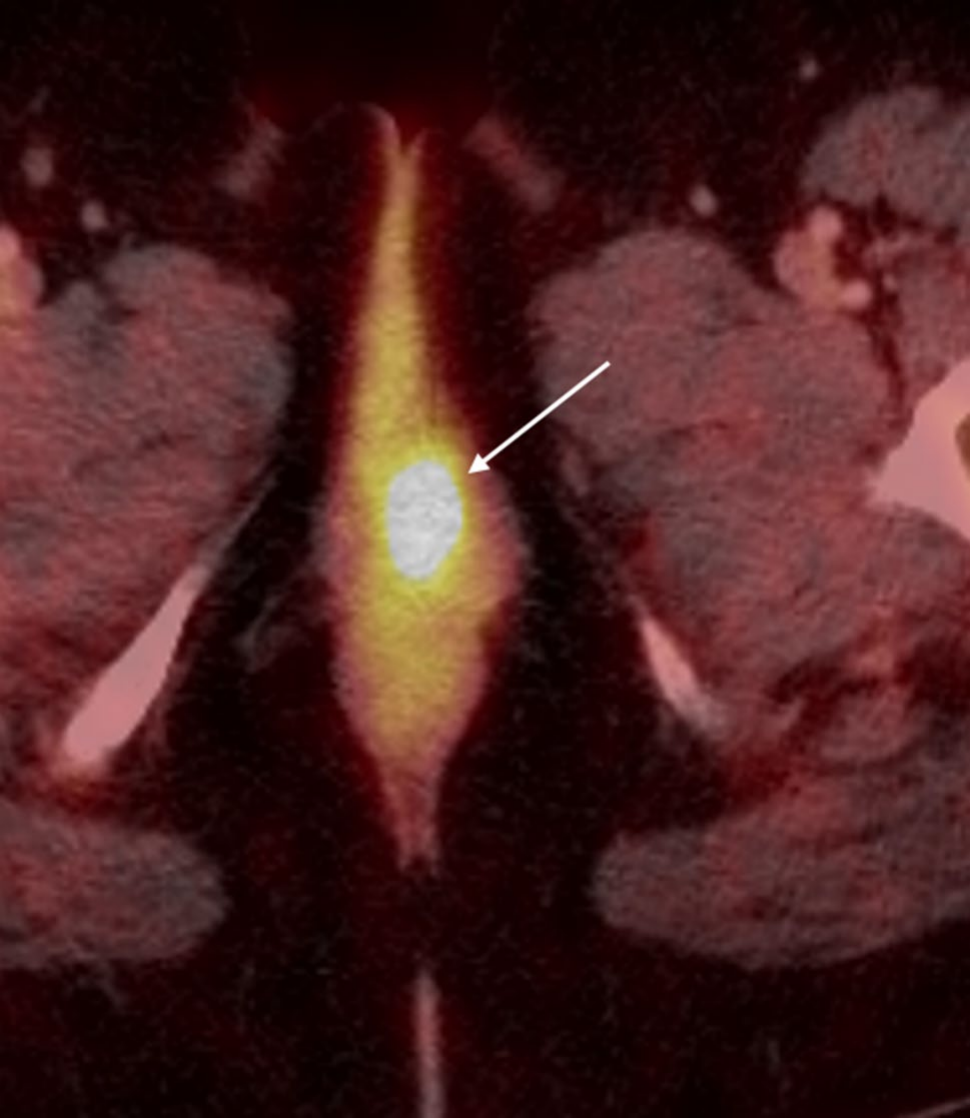
46-year-old woman with FIGO stage 1 vulvar adenoid cystic carcinoma of the left Bartholin gland. Axial PET-CT shows an intensely FDG-avid soft tissue mass (arrow). There was no evidence of lymphadenopathy or distant metastases (not shown). The pathologic staging was pT1b N0 M0. The mass was excised and the patient subsequently received radiation therapy with chemosensitization

FIGO stage 1 vulvar cancers are confined to the vulva or perineum and do not exhibit lymph node involvement or invasion of adjacent structures [21] (Fig. 2). FIGO stage 1A tumor is smaller than 2 cm and with less than 1 mm stromal invasion (T1a). FIGO stage 1B tumor has grown larger than 2 cm or with greater than 1 mm stromal invasion (T1b). On CT, FIGO stage 1 vulvar lesions may appear as vulvar thickening. On MRI, vulvar lesions appear to be T1 hypo-isotense and T2 iso-hyperintense and demonstrate contrast enhancement.

#### FIGO stage 2

**Fig. 3:**
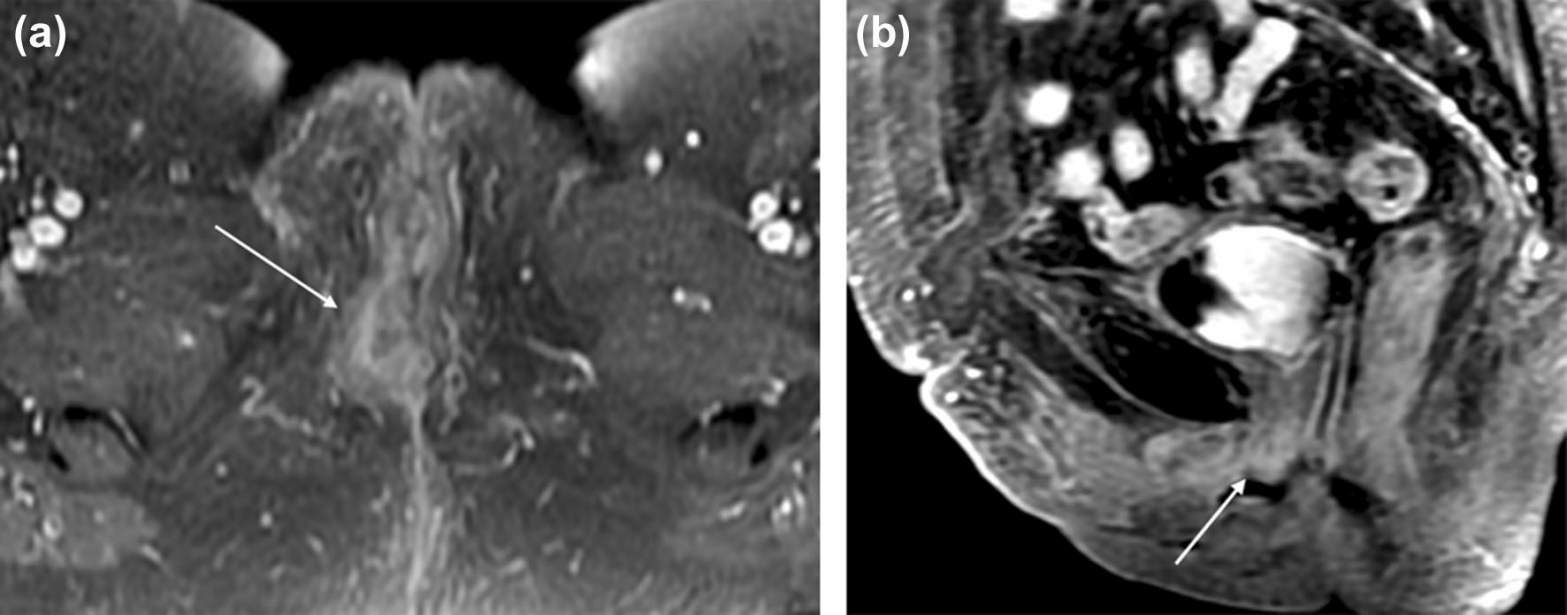
69-year-old woman with FIGO stage 2 squamous cell carcinoma of the vulva. **a** Axial T1-weighted fat saturation with contrast MR image of the pelvis at the level of the vaginal introitus demonstrates irregular thickening in the right labia (arrow). **b** Sagittal T1-weighted fat-saturated MR image with contrast demonstrates enhancing soft tissue surrounding the distal urethra (arrow), a feature of FIGO 2 stage tumor. Cystoscopy confirmed urethral involvement by tumor with meatal stenosis

FIGO stage 2 tumors can be any size and have partial invasion of adjacent structures including involvement of lower 1/3 urethra, lower 1/3 of vagina, or anus (T2), without metastatic nodal involvement (Fig. 3). MRI is the preferred modality to evaluate for extension of the primary vulvar lesion in locally advanced vulvar cancers. The architecture of the adjacent organs such as the urethra, vagina, and anal sphincter will be disrupted, and the involved portions will demonstrate contrast enhancement. Large necrotic tumors may demonstrate areas of marked T2 hyperintensity. For tumors larger than 2 cm or stage T1 and above, CT or PET-CT may be performed to evaluate for distant metastasis [22].

#### FIGO stage 3

**Fig. 4:**
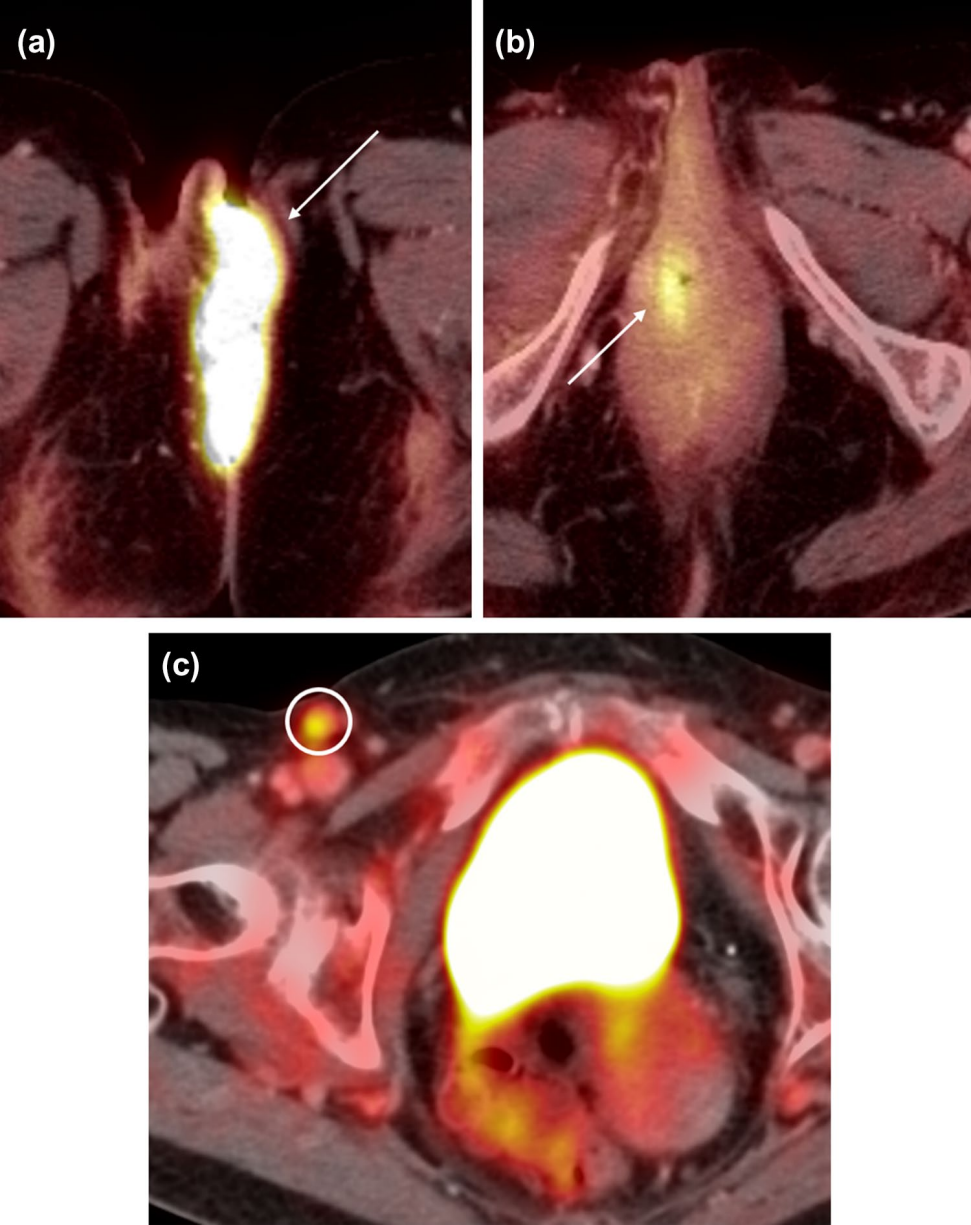
75-year-old woman with FIGO stage 3 vulvar cancer who presented with vaginal spotting. **a,b** Axial PET-CT demonstrates an intensely FDG-avid left vulvar mass (arrow in a) with extension to the right lateral vaginal wall (arrow in **b**). **c** FDG-avid right inguinal adenopathy is present (circle)

The hallmark of FIGO stage 3 vulvar cancers is lymph node involvement without distant metastases (Fig. 4). The tumor may be confined to the vulva (T1) or may extend into other structures including the lower urethra, lower vagina, or anus (T2). FIGO stage 3A vulvar cancer involves one inguinofemoral lymph node greater than 5 mm (N1) or it may involve 1 or 2 inguinofemoral lymph nodes less than 5 mm (N1). FIGO stage 3B vulvar cancer involves greater than 3 lymph nodes less than 5 mm (N2a) or the cancer involves 2 or more inguinofemoral lymph nodes greater than 5 mm (N2b). FIGO stage 3C vulvar cancers demonstrate presence of lymphadenopathy with extracapsular spread (N2c), generally pathologically determined. On MRI, metastatic nodal involvement appears to have characteristics such as irregular, rounded, spiculated morphology, short-axis diameter size greater than 10 mm, internal necrosis, loss of fatty hilum, and diffusion-weighted positivity [15]. Inguinal lymphadenopathy can be assessed on large field-of-view post-contrast T1-weighted images.

#### FIGO stage 4

**Fig. 5:**
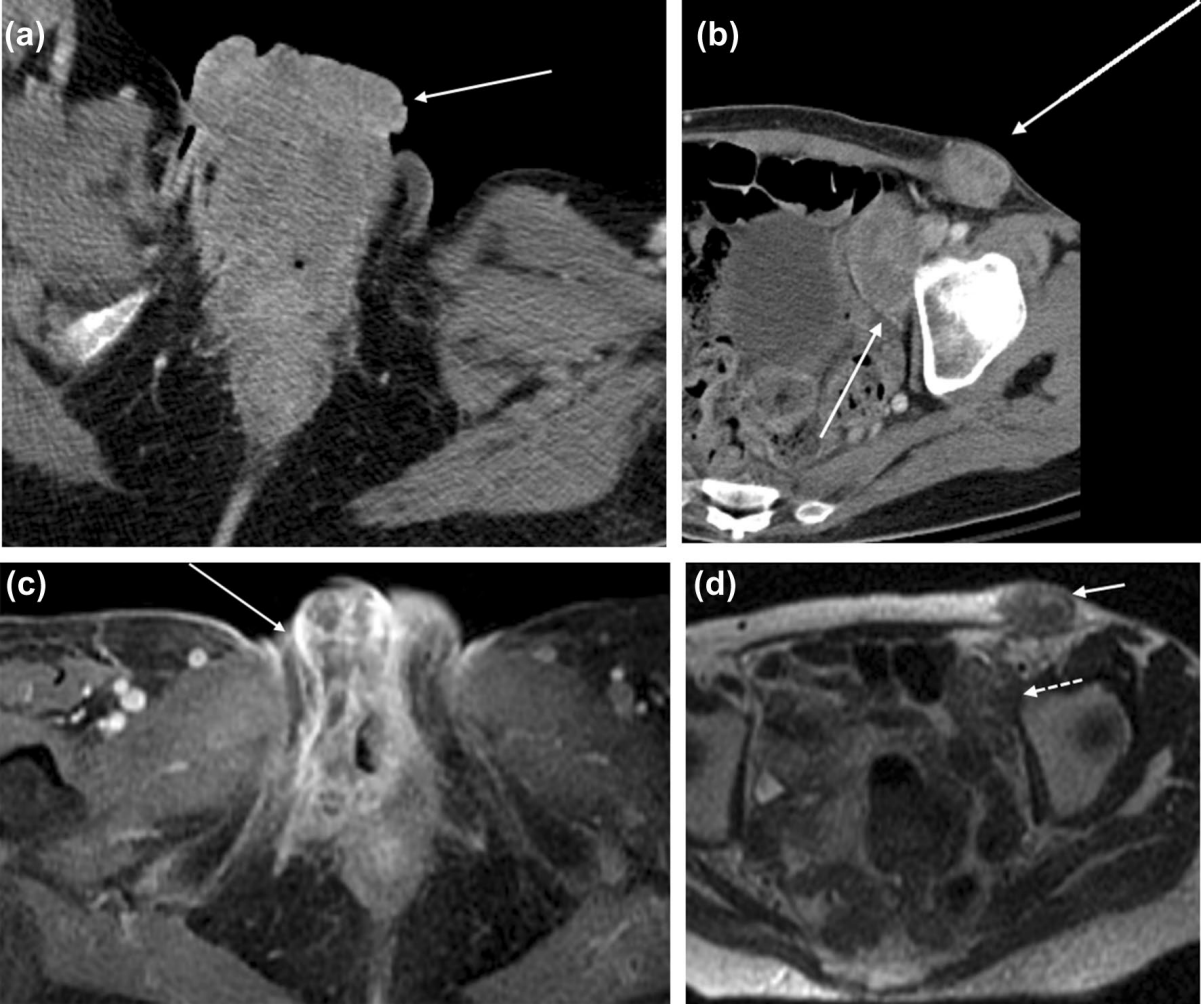
55-year-old woman with FIGO stage 4B squamous cell carcinoma of the vulva who presented with a malodorous vulvar mass. **a** Axial contrast-enhanced CT of the pelvis demonstrates a fungating vulvar mass (arrow). **b** Bulky left external iliac and inguinal lymphadenopathy was present (arrows). **c** Axial T1-weighted contrast-enhanced and **d** axial T2-weighted images show decreased size of the enhancing vulvar mass (solid arrow in **c**) and external iliac (dotted arrow) and inguinal (solid arrow in d) lymph nodes following radiation treatment

FIGO stage 4A vulvar cancers involves fixed or ulcerated inguinofemoral nodes (N3) or invasion of adjacent structures including the upper 2/3 of urethra, upper 2/3 of vagina, bladder, rectum, or pelvic bone (T3). FIGO stage 4B vulvar cancers involve distant pelvic lymph nodes (internal iliac, external iliac, common iliac, or paraaortic lymph nodes) or have distant metastases (M1), most commonly to the lungs (Fig. 5). Again, large field-of-view post-contrast T1-weighted images are also helpful to assess for pelvic adenopathy.

### Treatment

Treatment options for vulvar cancer involve a combination of surgery, radiation, and chemotherapy depending on the stage of disease. Surgery is the standard primary treatment of vulvar cancer. With early-stage vulvar cancer, surgery alone may be curative. Treatment of FIGO stage 1A tumor is wide excision. For FIGO stage 1B and 2 tumors, the treatment is radical local excision with sentinel lymph node dissection.

For FIGO stage 3 and 4A patients, treatment options include modified radical or radical vulvectomy with inguinal and femoral dissection followed by radiation treatment. With later-stage or non-resectable disease without major morbidities, neoadjuvant chemotherapy is the primary treatment. Consideration can be given for surgical removal of residual disease following chemoradiation. Newer management strategies start with neoadjuvant radiation therapy and/or chemoradiation of the primary lesion.

For stage 4B patients, there is no current standard treatment for advanced vulvar cancer. However, chemotherapy regimens that have been attempted include 5-fluorouracil (5-FU), cisplatin, mitomycin-C, or bleomycin [11]. Addition of palliative radiation may also be an option. Newer agents with targeted approaches toward immune signaling are promising therapies for HPV-driven vulvar malignancies [23].

### Survival/prognosis

Nodal status is the most important factor for survival, with an 86% 5-year survival rate in women who do not have lymph node involvement and 53% 5-year survival rate in women who have lymph node involvement [5].

The survival rate decreases as the FIGO stage increases. The FIGO staging and overall survival at 5 years is as follows: FIGO 1- 78%, FIGO 2- 59%, FIGO 3- 43%, and FIGO 4- 13%. The 5-year survival rate decreases to 19% when distant metastasis is identified [5].

Recurrence rates of vulvar SCC range from 30-50% within the first 2 years. Other prognostic features include tumor size, depth of invasion, and presence of metastatic disease [24].

### Post-treatment imaging

According to the NCCN (National Comprehensive Cancer Network), history and physical examination are recommended every 3 to 6 months for 2 years, followed by every 6 to 12 months for another 3 to 5 years, and then annually. Low-risk disease patients will have a longer follow-up interval whereas high-risk disease patients will have a shorter follow-up interval. Imaging is indicated by suspicious physical examination findings or symptoms of recurrence [25]. The purpose of post-treatment imaging includes assessing complications of treatment and identifying residual or recurrent tumor. A routine imaging surveillance strategy has not yet been established.

#### Complications

**Fig. 6:**
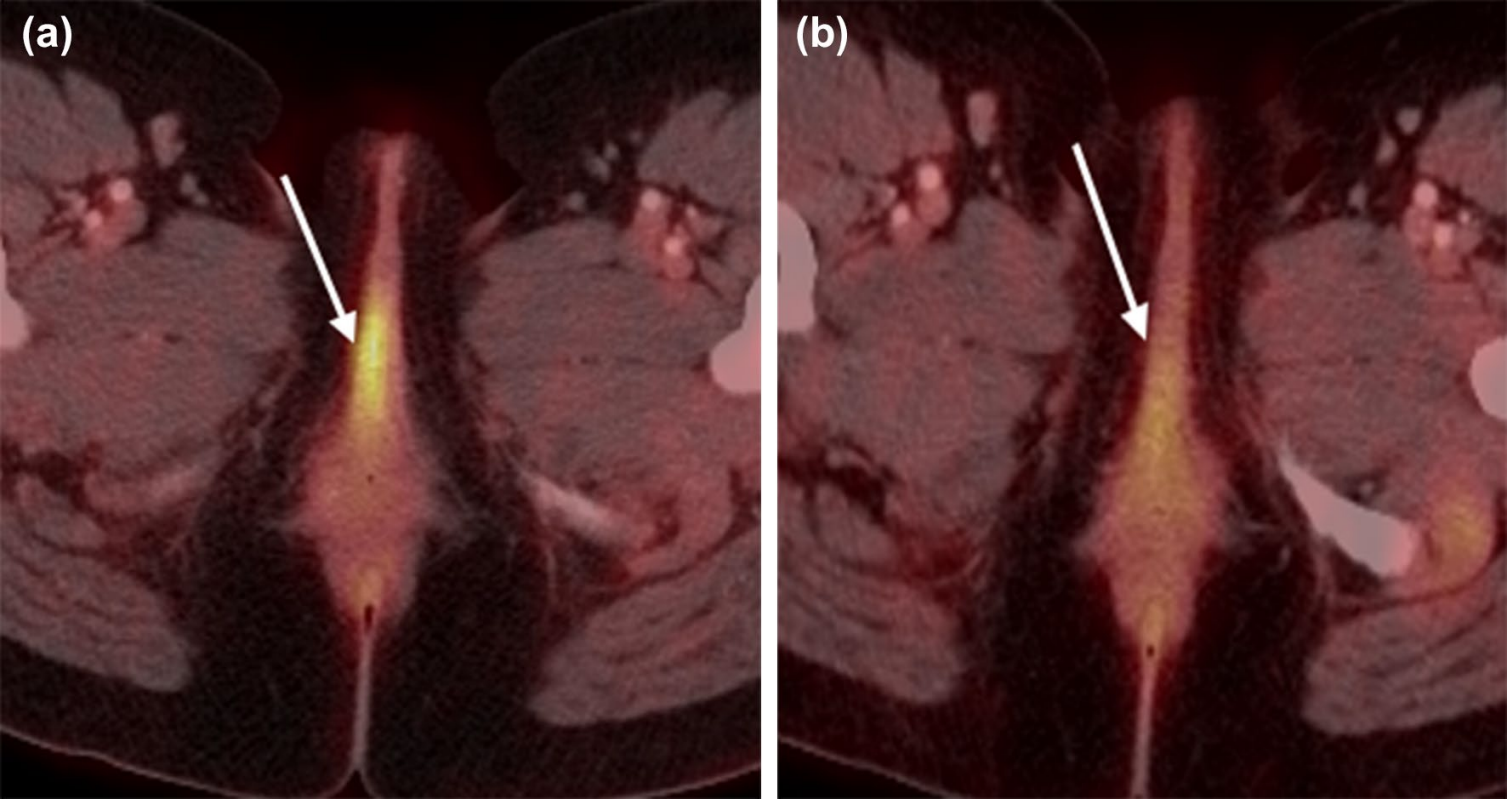
51-year-old woman with FIGO stage 1B squamous cell carcinoma of the vulva status post-excision. **a** Axial PET-CT image 3 weeks after surgery shows intense linear FDG uptake along the right vulva (arrow). **b** Axial PET-CT image six months later shows decreased FDG uptake. The previous findings reflect post-treatment inflammation reaction (arrow)

Post-treatment changes and complications after radical vulvar surgery include inflammatory reaction, pelvic floor prolapse, and urinary or stool incontinence (Fig. 6). Potential complications of groin lymph node dissection include lymphedema, lymphocele, wound infection or necrosis, hematoma, cellulitis, and hernia formation [26].

Early complications of radiation therapy include cellulitis, urethritis, vulvar edema, and diarrhea. Radiation-induced bone changes or fractures, myositis, fistula formation, and radiation-induced secondary tumors are late complications of radiation therapy. Fistulae between the bowel, vagina, or bladder can occur.

#### Recurrence

**Fig. 7:**
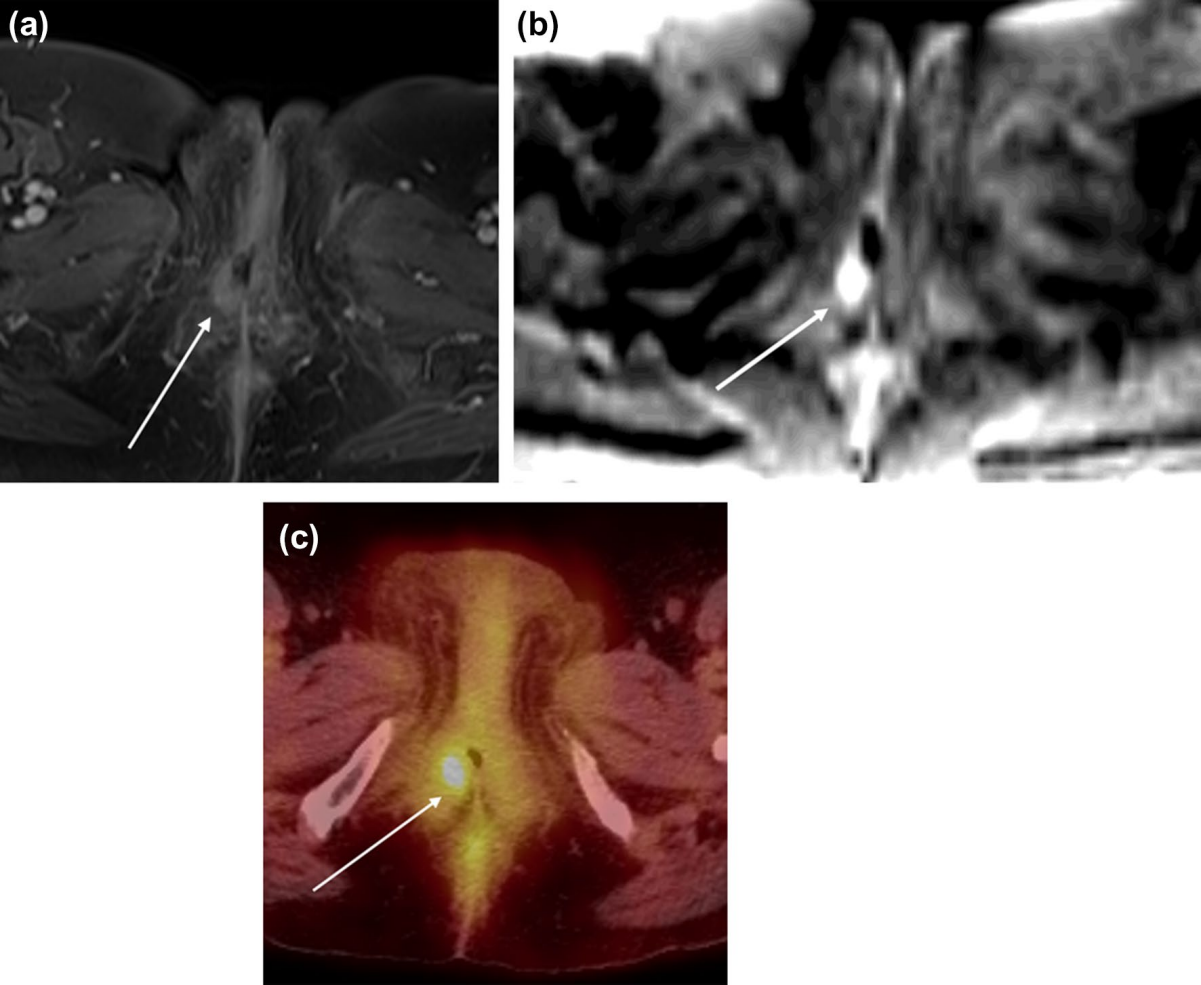
56-year-old woman with FIGO stage 4A SCC of the vulva status post-hysterectomy, bilateral oophorectomy, and radiation with recurrent disease 6 months later. **a** Axial T1-weighted contrast-enhanced and **b** DWI (b = 1000) MR images show a nodule measuring around 1 cm in the right vulva with enhancement and restricted diffusion (arrow). ADC image shows corresponding hypointensity (not shown). **c** Axial PET-CT image demonstrates soft tissue thickening in the right vulva with focal intense FDG uptake (arrow)

Local structures in the perineum serve as common sites of recurrence. Approximately 30% of recurrence can occur in the groin, which is associated with a worse outcome [27]. MRI can help delineate local recurrence whereas PET-CT can detect distant and nodal metastasis, particularly in the inguinal region with a 100% sensitivity and 89% specificity rate for positive inguinal nodes [28] (Fig. 7).

## Vaginal cancer

Approximately 5,400 patients are diagnosed with primary vaginal cancer in the USA each year [1]. Primary vaginal cancer constitutes about 2% of malignant neoplasms of the female genital tract. There is a high mortality rate with 30% of patients dying of the disease. The incidence of primary vaginal cancer increases with age with half of patients presenting after age 70. Risk factors for vaginal cancer include human papillomavirus, tobacco use, younger age at coitarche, multiple sexual partners, history of in situ or invasive cervical cancer, or prior pelvic radiation.

The most common clinical symptom of vaginal cancer is vaginal bleeding [29]. Other presenting symptoms result from local spread of disease, such as increased urinary frequency or tenesmus. Vaginal tumors typically present as a fungating mass, an ulcerating lesion, or annular constricting mass. However, approximately 10% of women eventually diagnosed with vaginal cancer are asymptomatic at the time of diagnosis, and their vaginal cancer is only detected from a Pap smear or physical exam.

At presentation, approximately 18% of women have stage 1 disease, 46% of women have stage 2 disease, and the remaining women have more advanced disease [30].

### Pathology

Similar to vulvar cancer, squamous cell carcinoma (SCC) is the most common histologic subtype and arises from the vaginal mucosa and frequently involves the proximal 1/3 of the vagina. Many SCC may be preceded by vaginal intraepithelial neoplasia (VAIN), which is defined as squamous cell atypia without invasion. SCC is most common in post-menopausal women with a 5-year survival of 54%.

In contrast, adenocarcinoma is more common in younger women and arises from glandular cells in the vagina such as the cells in the periurethral glands [31]. The most notable subtype of adenocarcinoma is clear cell adenocarcinoma, which is associated with in utero exposure to diethylstilbestrol, which is no longer commercially available. Adenocarcinoma is associated with an increased likelihood for lymph node and metastatic pulmonary involvement.

Melanoma arises from melanocytes that are present in the local epithelium and is overall a rare entity [32]. It usually occurs in the distal vagina, particularly on the anterior vaginal wall, and has the highest mortality rate of all vaginal malignancies with a 5-year survival of only 15% [31].

Metastatic spread or direct extension to the vagina from other cancers are more common than primary vaginal cancer, comprising over 80% of all vaginal tumors. The vagina is a common organ where endometrial, cervical, vulvar, ovarian, bladder, urethral, or rectal cancers can extend. Gestational trophoblastic disease can also spread to the vagina via lymphovascular spread. Breast and lung cancer can metastasize to the vagina as well, but this is rare.

### Anatomy

**Fig. 8:**
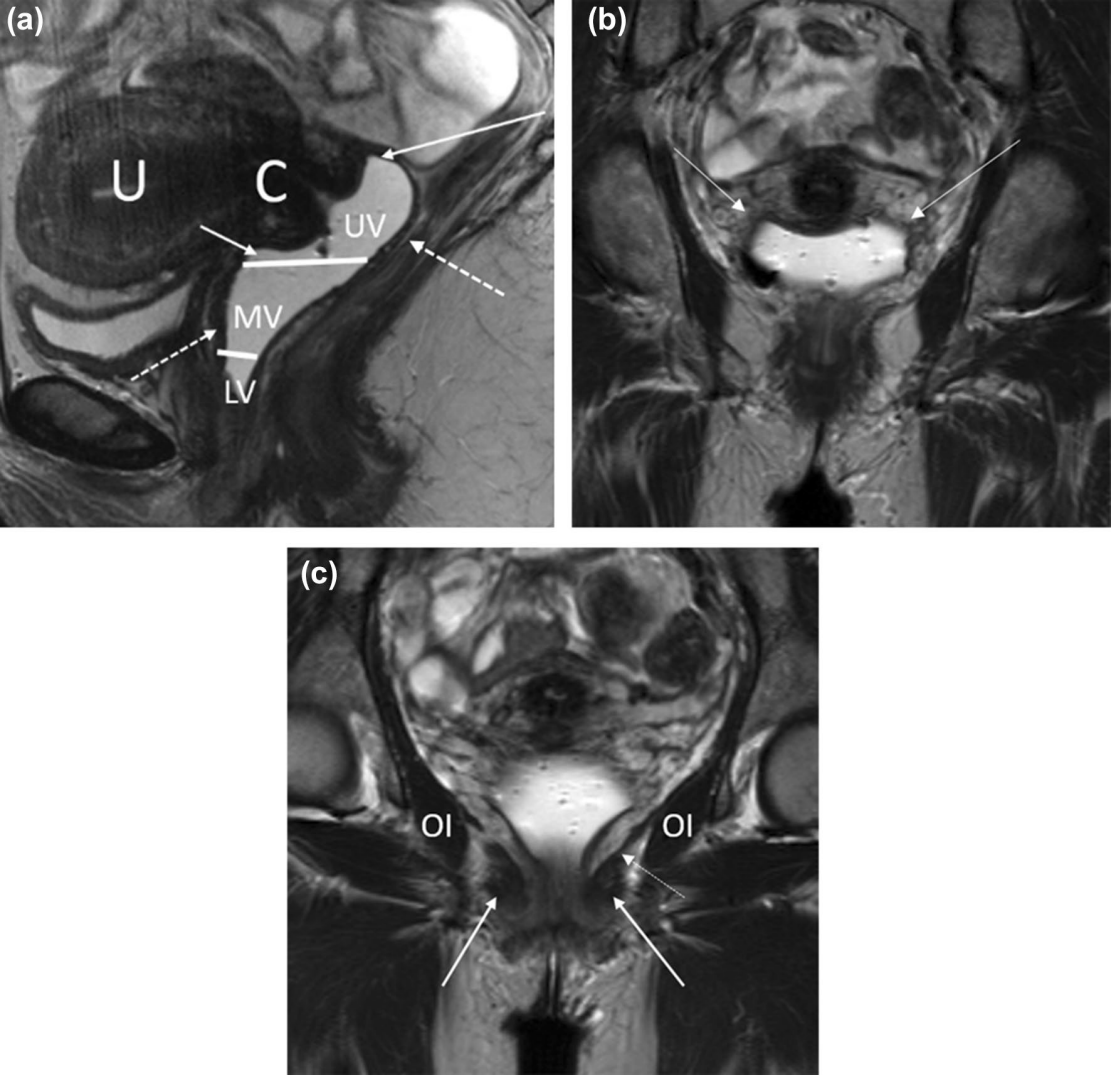
Relevant MR anatomy of the vagina. **a** Sagittal T2-weighted MR image of the vagina (distended with gel) shows the upper (UV), middle (MV), and lower (LV) thirds. The image also shows the anterior and posterior fornices (solid arrows), cervix (C), and uterus (U). The dotted arrows demonstrate the T2-hypointense muscularis layer of the vagina. **b** Coronal T2-weighted MR image of the vagina shows the lateral fornices of the vagina (solid arrows). **c** Coronal T2-weighted MR image through the vagina demonstrates the puborectalis (solid arrow), levator ani (dotted arrow), and obturator internus (OI)

The vagina is a fibromuscular cylindrical structure extending from the vulva to the distal cervix, measuring approximately 7 to 9 centimeters in length. The vaginal wall is composed of three layers: the mucosa, the muscularis, and the adventitia [33]. The vagina can be separated into three anatomic divisions. The upper third of the vagina borders are the anterior, posterior, and lateral vaginal fornices with lymphatic drainage into the external and internal iliac lymph nodes. The middle third of the vagina is posterior to the bladder base with lymph node drainage into the internal iliac chain. The lower third of the vagina is located inferior to the level of the bladder base and posterior to the urethra with lymph node drainage into the superficial inguinal nodes [34, 35] (Fig. 8).

### Patterns of spread

Vaginal tumors spread via direct extension into the surrounding pelvic structures including the paravaginal tissue, parametria, pelvic musculature, urethra, bladder, and rectum. Lymphatic spread of the vagina is complex and usually follows the division of the vagina. Hematogenous spread to the lung, liver, and osseous structures is rare and occurs in advanced stages.

### Imaging

#### CT/PET-CT

Similar to vulvar cancer, CT may be helpful in determining disease extent and nodal/metastatic involvement; however, it has lower sensitivity compared to MR and PET-CT. Three-dimensional CT reconstructions of the tumor can be used for treatment planning for radiation therapy.

Comparable to vulvar malignancies, PET-CT is useful for diagnosis of recurrent vaginal cancers (18). It is superior to CT at evaluating disease extent and nodal/metastatic involvement but less sensitive compared to MRI for local disease (18).

#### MRI

As with vulvar cancers, MRI is helpful to determine tumor size and extent due to its superior soft tissue resolution. MR is a reliable modality to detect primary and metastatic cancer with a reported accuracy of up to 82% and 92% for detecting locally recurrent and metastatic disease, respectively [36]. The muscularis of the vagina is represented as a T2-hypointense layer in the outer wall of the vagina. Vaginal gel can be introduced to allow for vaginal wall distention and better delineation of tumor size, location, and depth. A sample protocol for MRI imaging of vulvar and vaginal cancers is included (Table 3).

**Table 3:** MRI protocol of the female pelvis at our institution

Sequence type	Field of view (cm)	Slice thickness (mm)	Skip (mm)
Sagittal T2	34	5	1.5
Coronal T2	34	5	1.5
Axial T2	34	5	1.5
Sagittal T2 small FOV	17	3	0.6
Oblique Long-Axis T2 small FOV (with respect to the uterus)	17	3	0.6
Oblique Short-Axis T2 small FOV (with respect to the uterus)	17	3	0.6
Axial Diffusion-Weighted Imaging	34	6	2
Axial T1 Dixon Imaging	34	2.5	0
Axial T1 Fat Saturation PreContrast	34	1.6	0
Axial T1 Fat Saturation Post-Contrast	34	1.6	0
Coronal T1 Fat Saturation Post-Contrast	34	1.6	0

### FIGO classifications and imaging features

#### FIGO stage 1

**Fig. 9:**
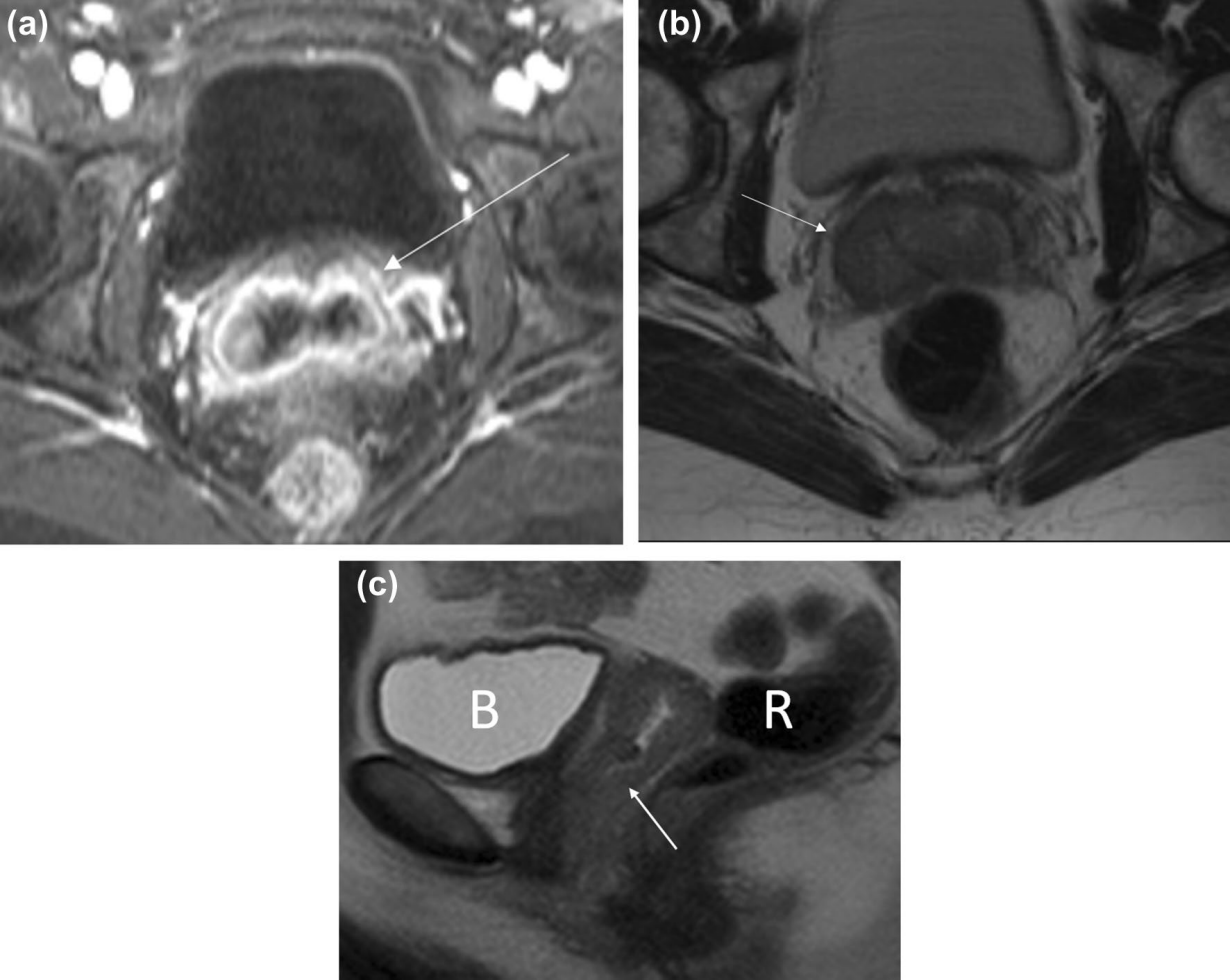
51-year-old woman with a history of cervical cancer who developed FIGO stage 1 squamous cell carcinoma of the vagina. **a** Axial T1-weighted fat-saturated contrast-enhanced MR image shows thickening of the vagina (arrow). **b** Axial T2-weighted MR image shows no paravaginal involvement (arrow) with an intact vagina. **c** Sagittal T2-weighted images show that the tumor is confined to the vaginal wall with no involvement of the rectum (R) or bladder (B)

In FIGO stage 1, the tumor is confined to the vaginal wall and its size can be smaller than 2 cm (T1a) or larger than 2 cm (T1b). On imaging, the tumor may have an imaging appearance of circumferential thickening of the vagina, as an ill-defined diffuse mass or as a bulky, lobulated mass [37] (Fig. 9). On MRI, vaginal tumors are usually T1 iso-hypointense and mildly T2 hyperintense relative to the vaginal wall. The tumors are best characterized on T2-weighted imaging and cause asymmetric abnormality of the vaginal contour. The tumor demonstrates early arterial enhancement on fat-suppressed T1-weighted dynamic contrast-enhanced imaging. On a T2-single-shot fast-spin echo (SSFSE) or T2-turbo spin-echo sequence, the T2-hypointense signal of the vaginal muscularis layer is intact. The T2-hyperintense paravaginal fat is uninvolved.

#### FIGO stage 2

**Fig. 10:**
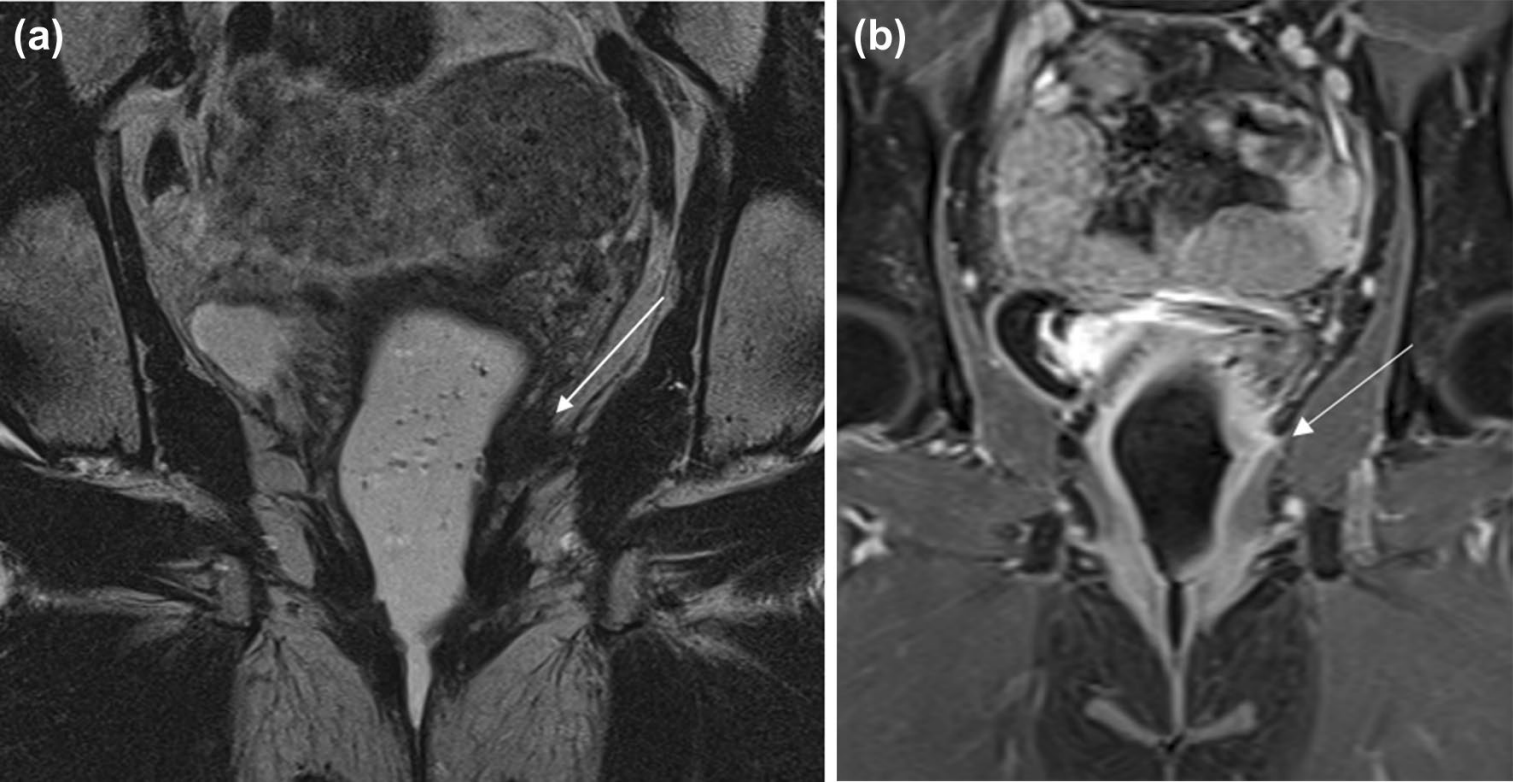
51-year-old woman with FIGO stage 2 squamous cell carcinoma of the vagina. **a** Coronal oblique long-axis T2-weighted image through the vagina shows a hypointense mass arising from the left vaginal wall (arrow) and extending into the paravaginal soft tissue. **b** Coronal T1-weighted post-contrast image shows enhancement within the paravaginal soft tissue (arrow)

FIGO stage 2 vaginal cancer is defined as extension beyond the vaginal wall and invasion into the paravaginal tissue but not involving the pelvic sidewall, regardless of size (Fig. 10). FIGO stage 2 encompasses tumor size less than 2 cm (T2a) and tumor size greater than 2 cm (T2b). On MRI, the T2-hypointense signal of the muscularis layer of the vaginal wall is disrupted. The coronal and sagittal planes are helpful in assessing the subtle disruption of the vagina.

#### FIGO stage 3

**Fig. 11:**
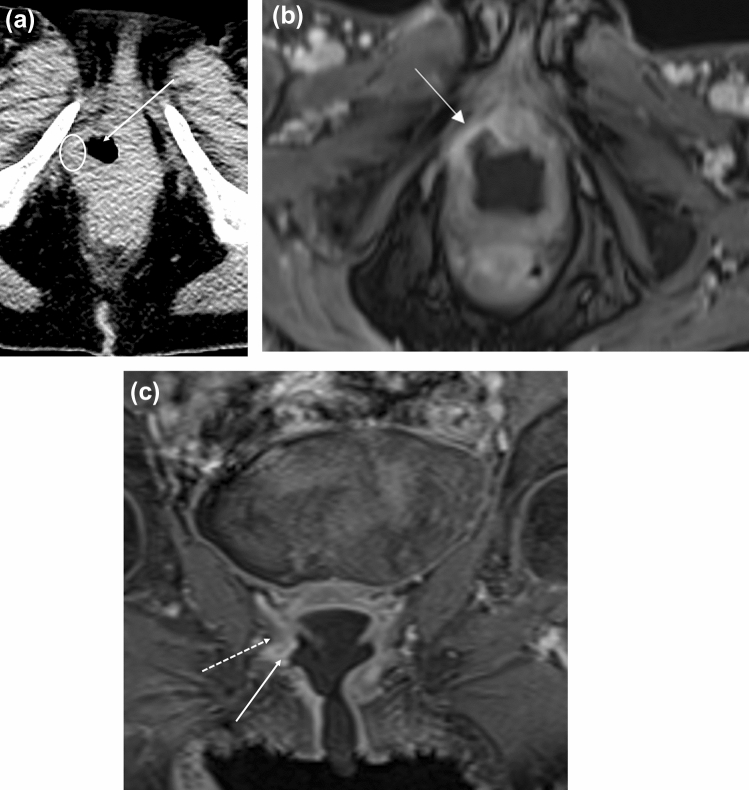
58-year-old woman with FIGO stage 3 squamous cell carcinoma of the vagina who was treated with chemoradiation. **a** Axial CT before treatment shows an ulcerated mass in the right vaginal wall (arrow). There is a subtle area of hypodensity representing the non-ulcerated portion of the mass (circle). **b** Axial T1-weighted contrast-enhanced MR image after treatment shows enhancing tissue (arrow) compatible with residual tumor. **c** Coronal T1-weighted contrast-enhanced MR image shows that the lesion (solid arrow) is predominantly infralevator in location but extends superiorly to involve the levator ani muscle (dotted arrow)

FIGO stage 3 vaginal cancer is characterized by pelvic sidewall invasion (T3) with involvement of the obturator internus, levator ani, piriformis muscles or iliac vessels, and/or hydronephrosis (Fig. 11). Axial and coronal images are helpful with identifying extent of pelvic sidewall invasion. FIGO stage 3 tumors may have also spread to local lymph nodes in the pelvis or inguinal regions including the perivisceral, inguinal, and internal and external iliac lymph nodes (N1) but not distant sites such as the common iliac or paraaortic lymph nodes [4]. On MRI, abnormal T2-hyperintense signal of the pelvic musculature may reflect edema or direct invasion into the musculature. An indirect sign of involvement is tethering of the adjacent structures and musculature.

#### FIGO stage 4

**Fig. 12:**
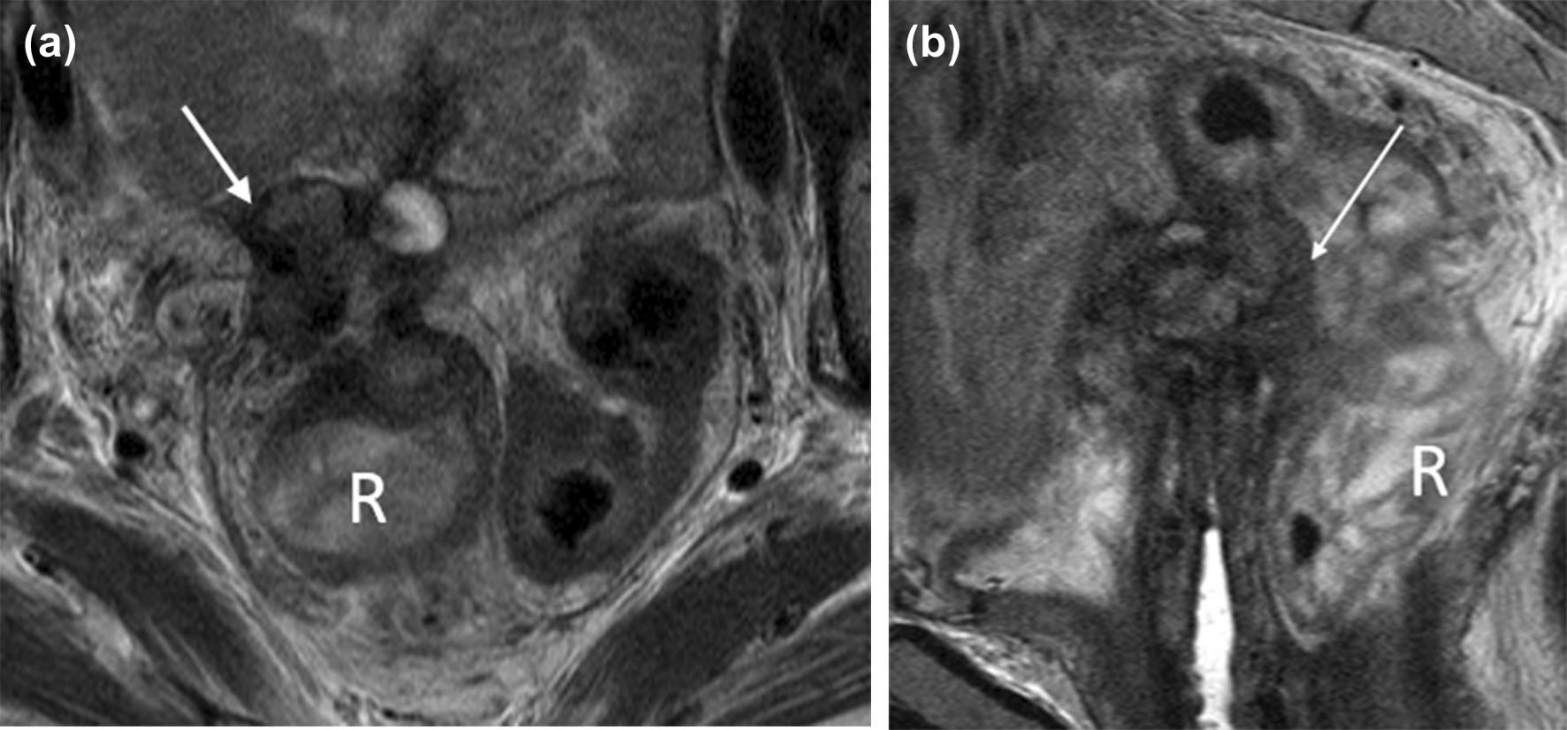
62-year-old woman with FIGO stage 4 vaginal cancer. **a** Axial T2-weighted MR image shows a complex soft tissue mass (arrow) with a cystic component at the superior edge of the vaginal cuff. The mass abuts several small bowel loops, with apparent tethering of surrounding structures. **b** Sagittal T2-weighted MR image shows that the mass invades the upper rectum (arrow). There is circumferential wall thickening of the upper rectum and sigmoid colon. R = rectum

In FIGO stage 4A disease, the tumor extends beyond the pelvis and exhibits bladder, urethra, or rectal invasion (T4) (Fig. 12). Bladder or rectal involvement is characterized by the disruption of the T2-hypointense bladder or rectal muscularis by the vaginal tumor and the complementary loss of fat planes between the vagina and these adjacent structures. Another suspicious imaging finding of bladder or rectal involvement is contour irregularity and nodularity along the bladder or rectal wall, respectively. Evaluation of these structures in multiple planes is useful with identifying invasion. Abnormal enhancing tumor involving the bladder or rectum can be a sign of invasion on contrast-enhanced T1-weighted images. A pitfall in determining bladder involvement is differentiating between peritumoral edema and inflammation from tumor invasion. Thus, in suspected cases of bladder invasion, cystoscopy is recommended for clinical confirmation [38]. Indirect imaging signs of bladder or rectal involvement is bladder distension from bladder outlet obstruction or rectal obstruction, respectively.

FIGO stage 4B disease demonstrates distant organ metastasis (M1). The disease has spread beyond the pelvis and may infiltrate the peritoneum and bowel loops. Lung, liver, and bone metastases are the most common distant organ metastases in FIGO stage 4B disease.

### Treatment

The stage and location of vaginal tumors determine the treatment strategy. In vaginal cancer, patients with early-stage cancer can be cured with surgical excision; however, radiation therapy alone may be an option as well. For stage 1 patients, treatment includes surgery including wide local excision or vaginectomy with or without lymph node dissection. Radiation therapy with external beam radiation therapy (EBRT) and/or combination of interstitial and intracavitary therapy can be considered at this stage. Stage 2 treatment consists of a combination of brachytherapy and EBRT. Stage 3 treatment options are external beam radiation therapy (EBRT) alone, or in combination with interstitial and/or intracavitary radiation. For patients with more advanced disease, chemoradiation is the predominant therapy. Stage 4a treatment includes a combination of interstitial, intracavitary, and external beam radiation therapy (EBRT). In stage 4B patients, the treatment is palliative radiation with or without chemotherapy [39].

### Survival/prognosis

Locoregional recurrence with involvement of the vaginal/paravaginal area and pelvic or inguinal lymph nodes occurs in approximately 25% of patients within 5 years [40]. Characteristics such as advanced stage and lower/posterior tumor location are correlated with higher rates of recurrence. Predictors for locoregional and metastatic recurrence include primary lesion larger than 5 cm, lower vaginal lesions including the middle and distal 1/3 of the vagina, and posterior vaginal wall lesions [41]. The 5-year survival rate for FIGO stage 1, 2, 3, and 4 cancer has been reported as 74, 54, 34, and 15%, respectively [42]. Lymph node involvement is important to identify as it correlates with 5-year survival.

### Post-treatment imaging

The purpose of post-treatment imaging includes assessing complications of treatment and identifying residual or recurrent tumor.

#### Complications

**Fig. 13:**
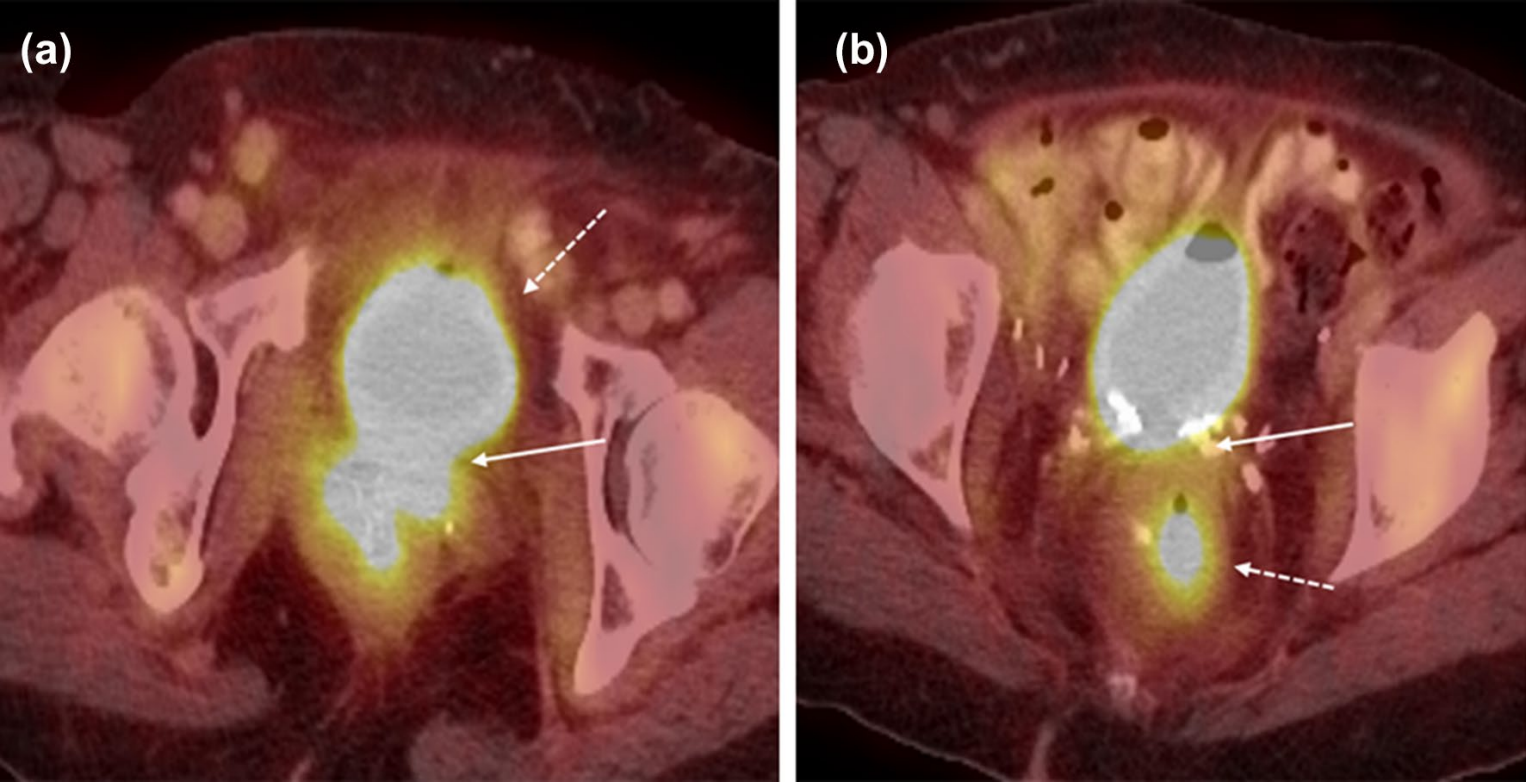
62-year-old woman with a history of FIGO stage 2 vaginal squamous cell carcinoma, status post-radiation therapy with vesicovaginal and colovaginal fistula. **a** Axial PET-CT image shows FDG-avid urine within the bladder (dotted arrow) extending into the vagina (solid arrow) and **b** rectum (dotted arrow)

Factors that increase the likelihood of complications include higher stage, larger tumor size, and higher radiation dose. The surrounding pelvic organs can be affected with rectovaginal fistula or vesicovaginal fistula since chemoradiation causes increased fibrosis, loss of soft tissue planes, and ischemic necrosis resulting in fistula formation (Fig. 13). Pelvic fistulas generally develop 1.5 years after treatment. The abnormal connections between the epithelial surfaces can best be delineated by MR imaging as a high signal intensity fistulous tract on T2-weighted imaging or direct contrast excretion through the fistulous tract on T1-weighted post-contrast imaging. Sagittal plane delayed T1 post-contrast sequences are particularly helpful for assessing pelvic fistulas. Other complications include cystitis, proctitis, and bowel strictures or perforation. Osseous complications like pelvic bone osteonecrosis or stress fractures can occur.

#### Imaging response/recurrence

**Fig. 14:**
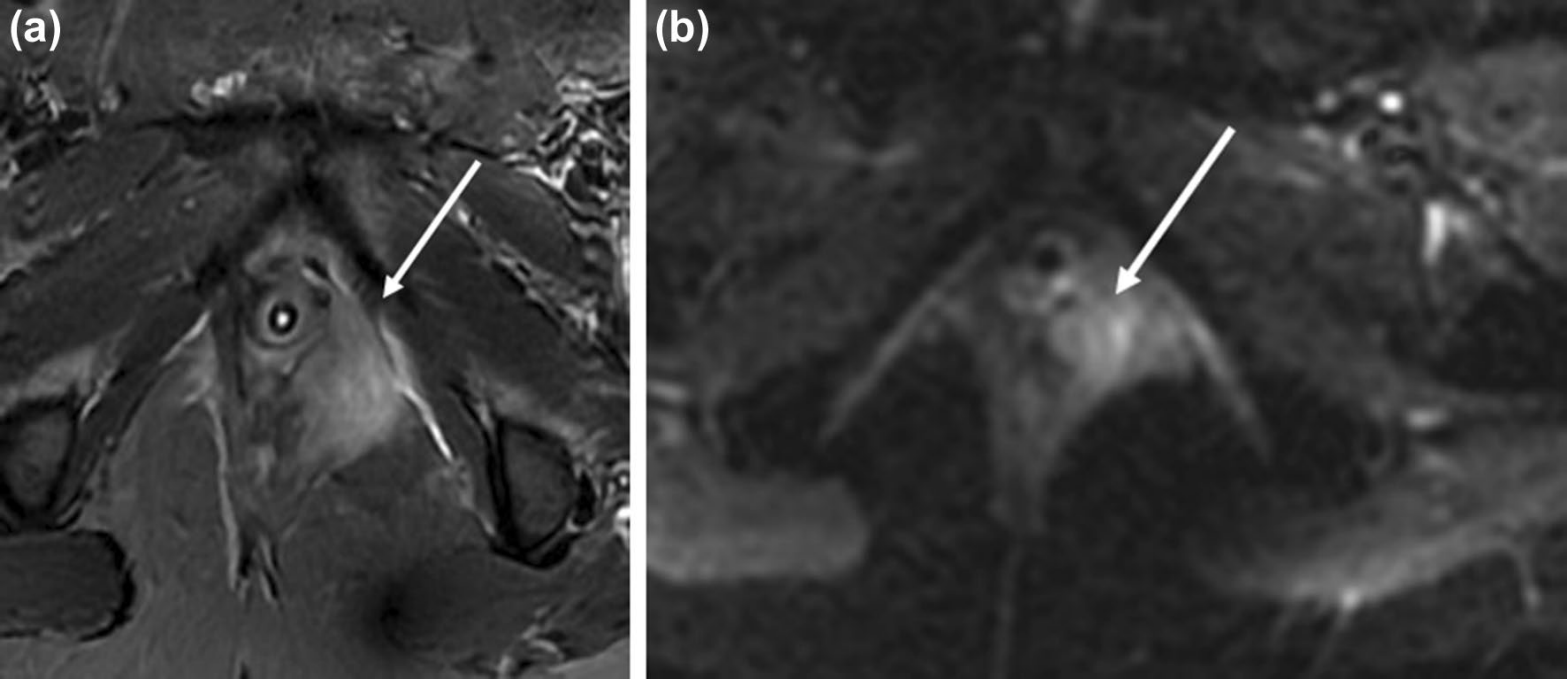
48-year-old woman with recurrent squamous cell carcinoma of the vagina. **a** Axial T2-weighted fat-saturated MR image shows a hyperintense mass within the left perineum causing mass effect on the left lateral vaginal wall (arrow) and extending into the left periurethral space. **b** DWI shows the lesion (arrow) with restricted diffusion

MR imaging is a useful imaging tool to help distinguish between tumor recurrence and post-treatment changes. In the time course between 0 and 6 months after treatment, the vaginal wall will be T2 hyperintense due to post-radiation mucosal and intramuscular edema, and it becomes challenging to distinguish between normal post-treatment changes and residual or recurrent disease. A reliable way to determine tumor response is to measure a decrease in tumor size. Imaging characteristics of residual or recurrent tumor will be T2 hyperintense and demonstrate early enhancement, whereas treated tumor or fibrosis/scar generally will not demonstrate early enhancement. Diffusion-weighted imaging (DWI) may be helpful in differentiating recurrent disease and post-treatment changes, with low and high ADC values suggestive of tumor and edema/inflammation, respectively [43] (Fig. 14).

Radiation-induced edema and other post-treatment changes typically resolve 6 months after treatment. At this time, any nodular or irregular T2-hyperintense thickening or enhancement, with or without restricted diffusion, would be suspicious for recurrent disease. Tissue fibrosis and scarring will appear T2 hypointense without enhancement. PET-CT will assist in evaluating for recurrent disease and distant metastases whereas MRI will better measure the extent of local tumor infiltration and tumor bulk.

## Conclusion

In the 2009 FIGO staging system, primary vulvar are staged surgically and clinically whereas primary vaginal cancers are staged clinically. Diagnostic imaging helps play a role in determining local disease extent since tailored surgical approaches and neoadjuvant chemoradiation therapy have been implemented to reduce morbidity. An understanding of the revised staging classification of vulvar and vagina carcinomas has direct relevance to disease management. Imaging is frequently obtained in the assessment of disease response and recurrence.
